# Effectiveness and Nephrotoxicity of Intravenous Polymyxin B in Chinese Patients With MDR and XDR Nosocomial Pneumonia

**DOI:** 10.3389/fphar.2020.579069

**Published:** 2021-02-05

**Authors:** Huihui Zeng, Zihang Zeng, Xianglong Kong, Hongliang Zhang, Ping Chen, Hong Luo, Yan Chen

**Affiliations:** ^1^ Department of Respiratory Medicine, The Second Xiangya Hospital, Central South University, Changsha, China; ^2^ Research Unit of Respiratory Diseases, Central South University, Changsha, China; ^3^ Hunan Centre for Evidence-based Medicine, Changsha, China; ^4^ Department of Respiratory Medicine, The First Hospital of Changsha, Changsha, China; ^5^ Department of Emergency Medicine, The Second Xiangya Hospital, Central South University, Changsha, China

**Keywords:** polymyxin B, effectiveness, nephrotoxicity, nosocomial pneumonia, drug-resistance

## Abstract

**Background:** Nosocomial pneumonia is a major health and economic burden globally. Multidrug-resistant (MDR) or extensively drug-resistant (XDR) Gram-negative bacteria are the most common causative pathogens in critically-ill patients. Polymyxin B is a salvage therapy for MDR Gram-negative pathogens; however, the current literature on its effectiveness and nephrotoxicity is limited, including in Chinese patients.

**Methods:** We retrospectively analyzed 107 patients with nosocomial pneumonia caused by MDR or XDR Gram-negative bacteria treated with intravenous polymyxin B (2–3 mg/kg/day). Renal function was evaluated on the day before commencement of polymyxin B therapy and on the third and 7 days of treatment. Univariate and multivariate analyses were conducted to determine risk factors for the effectiveness and nephrotoxicity of polymyxin B. Sixty-seven (62.6%) and sixty-five (60.7%) patients had favorable clinical and microbiological responses, respectively. Acute physiology and chronic health evaluation II (APACHE II) scores, cardio-pulmonary resuscitation (CPR) history, numbers of pathogens per patient and a favorable microbiological response were independently associated with favorable clinical outcomes of polymyxin B treatment in Chinese patients with MDR or XDR nosocomial pneumonia. Initial renal dysfunction was not associated with late nephrotoxicity (on day 7), although early nephrotoxicity (on day 3) was independently associated with late nephrotoxicity (OR = 39.43, 95% CI 7.64–203.62, *p* = 0.00).

**Conclusion:** Our findings support polymyxin B treatment for MDR and XDR pneumonia, with the severity of disease and polymicrobial infection being risk factors for a poor clinical outcome. Nephrotoxicity following 3 days of polymyxin B treatment was found to be a reliable risk factor for later nephrotoxicity.

## Introduction

Polymyxins were approved for clinical use in the late 1950s (Tsuji et al., 2019), but were abandoned in the 1970s mainly due to nephrotoxicity and neurotoxicity (Kelesidis and Falagas 2015; Tsuji et al., 2019). However, Gram-negative bacilli, especially multidrug-resistant (MDR), extensive drug-resistant (XDR) and prodrug-resistant (PDR) pathogens, have become increasingly prevalent in nosocomial pneumonia (Kalil et al., 2016). Given this situation, the polymyxins have been revived as a salvage therapy for otherwise untreatable Gram-negative bacterial infections (Tacconelli et al., 2018; Tsuji et al., 2019).

Only polymyxin B and colistin are available in the clinic. Unlike colistin, which is administrated as an inactive prodrug (colistimethate sodium), polymyxin B is administered as its active form (Tsuji et al., 2019). As a consequence, intravenous use of polymyxin B can achieve efficacious plasma concentrations more rapidly than can colistin. A number of studies have demonstrated that polymyxin B has acceptable efficacy against infections caused by nosocomial MDR Gram-negative bacteria (Falagas et al., 2005; Li et al., 2006; Zavascki et al., 2007; Thomas et al., 2019). Polymyxin B is not significantly eliminated through the kidneys, and several clinical pharmacokinetic (PK) studies have demonstrated that its clearance is not associated with renal function (Sandri et al., 2013; Thamlikitkul et al., 2017). Therefore, it was assumed that the incidence and severity of acute kidney injury (AKI) associated with polymyxin B treatment would be limited. Nonetheless, the incidence of polymyxin B associated nephrotoxicity varies widely in the literature (Zavascki et al., 2017), due in part to the different definitions of nephrotoxicity applied, the wide range of polymyxin dosage regimens employed, and heterogeneities of patients (Tsuji et al., 2019). Several studies suggest that kidney dysfunction at baseline is a predictive factor for nephrotoxicity after intravenous polymyxin B in patients (Rigatto et al., 2015; Crass et al., 2017; John et al., 2018).

Severe infections (e.g., serious pneumonia or sepsis) can induce kidney dysfunction, a portion of which might be reversed by clearing the infection (Kalil et al., 2016; Rello et al., 2018). Thus, it might be difficult to differentiate between polymyxin B-associated AKI and infection-induced AKI without sufficient anti-infective treatment. Given antibiotic treatment less than 48 h is insufficient for pneumonia (Kalil et al., 2016), we speculated that AKI following long-term polymyxin B use represents the true level of drug-induced nephrotoxicity. As polymyxin B has only been available in Chinese hospitals since January 2018, there is limited data regarding its use in Chinese patients. Therefore, we conducted this retrospective study to assess the effectiveness and safety of polymyxin B in Chinese HAP and VAP patients.

## Methods

### Study Design

This study was approved and supervised by the Medical Research Ethics Committee of the Second Xiangya Hospital, Central South University (LYF2020059). This retrospective cohort study was performed at a 3500-bed tertiary teaching hospital, the Second Xiangya Hospital of Central South University, Changsha, in mid-southern China. The hospital records database was searched for all adult patients with MDR or XDR HAP and VAP who received intravenous polymyxin B for more than 48 h from January 1, 2018 to May 31, 2019. Written informed consent was waived. The diagnoses of MDR or XDR HAP and VAP were confirmed by two pulmonologists following the 2016 clinical practice guidelines of the Infectious Diseases Society of America and the American Thoracic Society (Kalil et al., 2016). Patients were excluded if they were younger than 14 years old, received polymyxin B therapy for <2 days, or causative MDR and XDR pathogens were not found.

**FIGURE 1:**
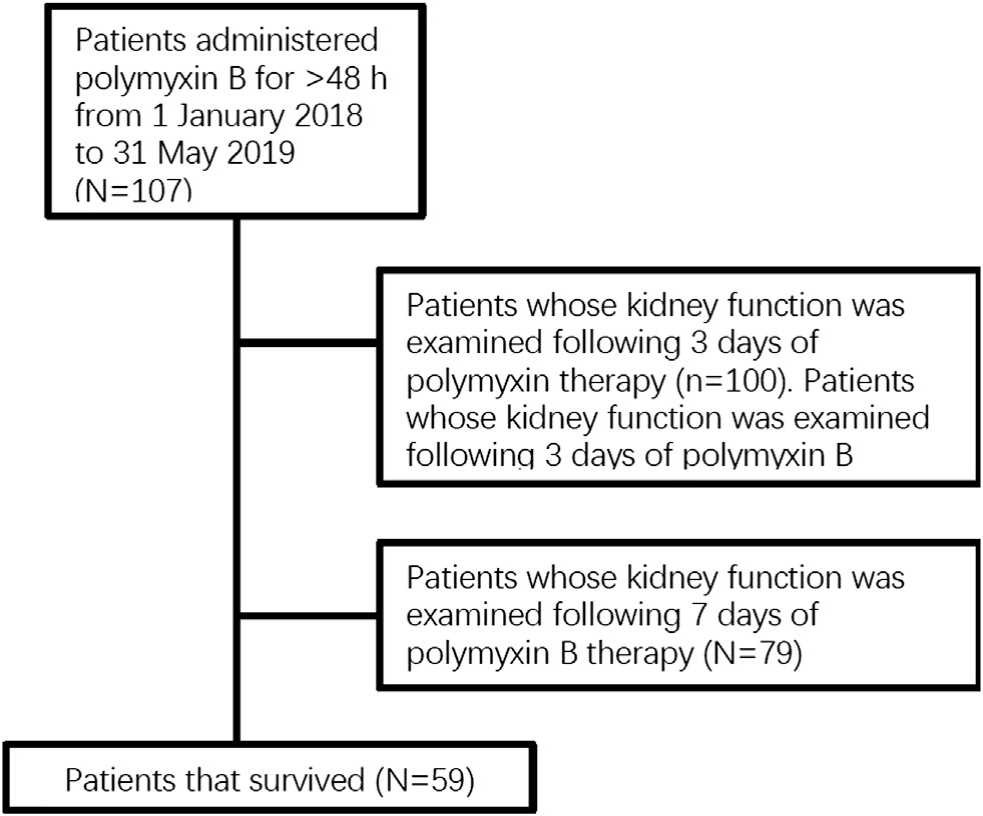
Flow chart of the number of eligible patients.

The following medical information was examined: demographics, comorbidity and concomitant diseases, duration of hospitalization and intensive care unit (ICU) stay, incidence of renal dysfunction, mortality, and use of concurrent treatments. Attributive variables of effectiveness were identified by comparison of clinically favorable and unfavorable outcomes in patients, and risk factors for nephrotoxicity were identified by comparison of patients with and without nephrotoxicity on different days of the treatment Figure 1.

### Microbiological Examination

Pathogen isolation and semi-quantitative culture (4-quadrant streaking) were achieved using deep sputum, endotracheal aspirate or bronchoalveolar lavage fluid specimen with an automated identification and susceptibility testing system (Phoenix100, BD, United States). The antibiotic susceptibility of microorganisms was determined via broth microdilution (Tsuji et al., 2019; Quintanilha et al., 2019; European Committee on Antimicrobial Susceptibility Testing, 2016).

### Variables and Definitions

To evaluated comorbidities in patients, the Charlson comorbidity index (CCI) was calculated as previous study (Charlson et al., 1994). Patient with GFR <60 ml/min was defined as kidney injury in baseline condition (Levin et al., 2013). Increased creatinine (Cr) was calculated by subtracting the creatinine level before polymyxin B use from the creatinine level after use. According to RIFLE criteria (Kellum et al., 2008), an increased Cr concentration of >1.5 fold or a glomerular filtration rate (GFR) decrease of >25% was defined as the risk stage of renal function, and an increased Cr concentration of >2 fold or a GFR decrease of >50% was defined as renal injury. Increased Cr concentration of >3.3 fold or a GFR decrease >75% was defined as renal failure. Early nephrotoxicity was defined as AKI on the third day of polymyxin B use, whereas late nephrotoxicity was defined as AKI on the seventh day of use.

Clinical outcomes were confirmed by two independent pulmonologists, and a favorable clinical response was defined as a complete or partial recovery of symptoms and signs at the end of polymyxin B treatment (Furtado et al., 2007). To define clinical outcomes, the following symptoms and signs were recorded: fever, leukocytosis and leukocytopenia, use of vasopressors, improvement of PaO_2_/FiO_2_ parameters and chest radiographs. An unfavorable clinical response was defined as persistence or worsening of symptoms and signs during polymyxin B treatment. Microbiological outcomes were evaluated by repeated microbiological cultures at the end of treatment. A favorable microbiological response was defined as a decreased quantity or clearance of causative pathogens, according to the results of microbiological semi-quantitative culture.

A patient’s baseline condition was assessed based on white blood cell (WBC), hemoglobin, platelet, albumin, alanine aminotransferase (ALT), aspartate transaminase (AST), total bilirubin (TBIL), Cr, blood urea nitrogen (BUN) and kidney function evaluations prior to polymyxin B administration.

### Statistical Analysis

Student’s *t*-test was conducted for continuous variables between groups. The χ^2^ or Fisher exact test was performed for categorical variables. Logistic regression models were built after univariate analysis. Variables found to be significantly associated with outcome and nephrotoxicity in the univariate analyses were entered in the multivariate backward logistic regression models. A two-tailed *p*-value < 0.05 was considered statistically significant. SPSS v. 10.0 (SPSS Inc., Chicago, United States) was employed for the statistical analysis.

## Results

This study included 107 cases of nosocomial pneumonia treated with intravenous polymyxin B due to infection caused by MDR and XDR bacilli. The mean age of all patients was 58.8 ± 17.3 years (range 17–91 years), and the total number of male patients was 75 (70.1%) (Table 1). The average APACHE II score was 16.3 ± 8.3 and the overall all-cause mortality and rate of mechanical ventilation were 44.9% (48 patients) and 73.8% (79 patients), respectively (Table 1). The high APACHE II score and mortality indicate the severity of disease in this patient population. In total, 101 patients (94.4%) were administrated intravenous polymyxin B at 2.5–3 mg/kg/day, whereas six patients (5.6%) were administrated 2 mg/kg/day (Table 1). Sixty-seven (62.6%) and sixty-five (60.7%) patients had favorable clinical and microbiological responses, respectively. The culture of responsible pathogens reveals that there were five fungal infection patients underlying with bacterial infection, suggesting a possibility of polymicrobial infection in MDR and XDR HAP patients. On the third day of polymyxin B treatment, the Cr and BUN levels of 100 patients were examined with 24 patients (24.0%) diagnosed with nephrotoxicity (risk stage 18, injury stage 4, failure stage 2). On the seventh day of polymyxin B treatment, kidney function tests were performed on 79 patients, 25 of whom (31.6%) were diagnosed with nephrotoxicity (risk stage 21, injury stage 3, failure stage 1). All of the 79 patients with 7-days treatment had been tested following 3 days of treatment, with 16 (20.3%) of these patients having been diagnosed with early nephrotoxicity on day 3. There was a significant difference between early and late polymyxin B associated nephrotoxicity rate in patients whose kidney function was tested on both day 3 and 7 (*p* = 0.00). In addition, the analysis of CCI showed that there was no significant difference between favorable and unfavorable clinical outcome groups, or AKI and non-AKI groups (Tables 1–3).

**TABLE 1:** Characteristics of cohorts and univariate analysis for clinical response to pneumonia.

	Entire cohort	Favorable clinical response	Unfavorable clinical response	*p*-value
	N = 107 (mean ± SD, or n (%))	N = 67 (mean ± SD, or n (%))	N = 40 (mean ± SD, or n (%))	
**Demographics**				
Age (years)	58.8 ± 17.3	57.3 ± 15.7	61.4 ± 19.8	0.26
Gender (male)	75 (70.1)	31 (46.3)	28 (70.0)	0.02
Weight (kg)	63.5 ± 8.3	63.2 ± 8.7	64.2 ± 7.7	0.51
APACHE II scores	16.3 ± 8.3	15.1 ± 7.7	22.5 ± 7.2	0.00
CPR history	19 (17.8)	6 (9.0)	13 (32.5)	0.00
ICU admission	96 (89.7)	58 (86.6)	38 (95.0)	0.16
Duration of ICU stay days since polymyxin B treatment	26.9 ± 19.8	26.6 ± 18.0	27.4 ± 22.6	0.84
Hospitalization days	39 ± 29.3	40.5 ± 26.4	36.4 ± 33.8	0.49
**Comorbidities**				
CCI	4.2 ± 2.9	4.1 ± 2.8	4.6 ± 3.2	0.17
Malignant disease	15 (14.0)	8 (11.9)	7 (17.5)	0.42
Hematological malignant disease	5 (4.7)	1 (1.5)	4 (10.0)	0.04
Pulmonary diseases	24 (22.4)	14 (20.9)	10 (25.0)	0.62
Heart Diseases	39 (36.4)	25 (37.3)	14 (35.0)	0.81
Diabetes	20 (18.7)	11 (16.4)	9 (22.5)	0.44
Transplantation history	17 (15.9)	14 (20.9)	3 (7.5)	0.07
Surgery	35 (32.7)	24 (35.8)	11 (27.5)	0.38
**Invasive procedures**				
Tracheotomy and intubation	79 (73.8)	43 (64.2)	36 (90.0)	0.00
Tracheotomy	32 (29.9)	17 (25.4)	15 (37.5)	0.19
Intubation	47 (43.9)	26 (38.8)	21 (52.5)	0.17
Central venous catheter	52 (48.6)	33 (49.3)	19 (47.5)	0.86
**Responsible pathogen**				
*Klebsiella pneumoniae*	39 (36.4)	21 (31.3)	18 (45.0)	0.28
*Acinetobacter baumannii*	63 (58.9)	39 (58.2)	24 (60.0)	0.86
*Pseudomonas eruginosa*	31 (29.0)	17 (25.4)	14 (35.0)	0.29
*Stenotrophomonas maltophilia*	4 (3.7)	2 (3.0)	2 (5.0)	0.60
*Escherichia coli*	3 (2.8)	0 (0.0)	3 (7.5)	0.02
Fungus	5 (4.7)	0 (0.0)	5 (12.5)	0.00
Numbers of pathogens per patient	1.3 ± 0.6	1.2 ± 0.4	1.8 ± 0.8	0.00
**Culture and susceptibility**				
Favorable microbiological response	65 (60.7)	54 (80.6)	11 (27.5)	0.00
Microorganism Clearance	26 (24.3)	24 (35.8)	2 (5.0)	0.00
Polymyxin B MIC ≤0.5 mg/L before treatment	55 (51.4)	40 (59.7)	15 (37.5)	0.03
**Polymyxin B treatment**				
Total dosage (mg)	1,157.4 ± 1,272.3	1,315.1 ± 1,517.0	893.3 ± 625.7	0.10
2 mg/kg/day	6 (5.6)	5 (7.5)	1 (2.5)	0.28
2.5–3 mg/kg/day	101 (94.4)	62 (92.5)	39 (97.5)	0.28
Load of 2–2.5 mg/kg as first dose	30 (28.0)	19 (28.4)	11 (27.5)	0.92
Duration days	10 ± 5.3	11.3 ± 5.6	7.8 ± 3.8	0.00
Nebulization and intravenous use	3 (2.8)	1 (1.5)	2 (5.0)	0.29
Polymyxin B monotherapy	2 (1.9)	1 (1.5)	1 (2.5)	0.71
Concomitant with *β*-lactam	42 (39.3)	27 (40.3)	15 (37.5)	0.77
Concomitant with carbapenem	29 (27.1)	22 (32.8)	7 (17.5)	0.08
Concomitant with tigecycline	39 (36.4)	21 (31.3)	18 (45.0)	0.16
**Underline condition (before polymyxin B treatment)**				
WBC (×10^9^/L)	11.1 ± 6.7	10.9 ± 6.0	11.3 ± 7.7	0.80
Hemoglobin (g/L)	88.1 ± 22.2	91.4 ± 23.1	82.7 ± 19.5	0.05
Platelets (×10^9^/L)	188.4 ± 134.8	217.8 ± 146.3	139.3 ± 96.2	0.00
Albumin (g/L)	33.1 ± 5.6	33.2 ± 5.4	32.9 ± 6.1	0.75
ALT (µ/L)	54.3 ± 94.6	60.7 ± 113.1	43.5 ± 50.2	0.37
AST (µ/L)	58.2 ± 59.9	49.9 ± 50.3	72.1 ± 71.9	0.37
TBIL (µmol/L)	43.1 ± 79.7	35.5 ± 63.0	55.8 ± 101.4	0.05
Cr (µmol/L)	124.2 ± 125.9	120.1 ± 120.6	130.9 ± 135.6	0.67
Kidney injury	28 (26.2)	16 (23.9)	12 (30.0)	0.04
**Outcome**				
Mortality	48 (44.9)	13 (19.4)	35 (87.5)	0.00
Early nephrotoxicity (on the 3rd day of polymyxin B treatment)	24/100 (24.0)	13/64 (20.3)	11/36 (30.6)	0.25
Late nephrotoxicity (on the 7th day of polymyxin B treatment)	25/79 (31.6)	15/57 (26.3)	10/22 (45.5)	0.10

APACHE; acute physiology and chronic health evaluation, CPR; cardio-pulmonary resuscitation, ICU; intensive care unit, CCI; Charlson comorbidity index, WBC; white blood cell, ALT; alanine aminotransferase, AST; aspartate transaminase, TBIL; total bilirubin, Cr; creatinine.

**TABLE 2:** Univariate analysis for early nephrotoxicity.

	AKI on the third day of treatment	Non-AKI on the third day of treatment	*p-*value
	N = 24 (mean ± SD, or n (%))	N = 76 (mean ± SD, or n (%))	
**Demographics**			
Age (years)	60.6 ± 15.7	56.4 ± 18.7	0.78
Gender (male)	17 (70.8)	51 (67.1)	0.73
APACHE II scores	19.1 ± 8.6	18.1 ± 9.0	0.61
CPR history	6 (25.0)	12 (15.8)	0.31
ICU Admission	22 (91.7)	67 (88.2)	0.63
Hospitalization days	32.3 ± 23.6	42.2 ± 31.4	0.16
Favorable clinical response	13 (54.2)	51 (67.1)	0.25
**Comorbidities**			
CCI	4.4 ± 2.8	4.4 ± 3.0	0.94
Malignant disease	2 (8.3)	13 (17.1)	0.29
Pulmonary diseases	3 (12.5)	18 (23.7)	0.24
Heart Diseases	12 (50.0)	26 (34.2)	0.17
Diabetes	3 (12.5)	16 (21.1)	0.35
Kidney disease	10 (41.7)	23 (30.3)	0.30
Transplantation history	3 (12.5)	14 (18.4)	0.50
Kidney transplantation	3 (12.5)	8 (10.5)	0.79
Surgery	7 (29.2)	27 (35.5)	0.57
**Polymyxin B treatment**			
Total dosage (mg)	1,478.2 ± 756.2	1,362.8 ± 995.8	0.00
2 mg/kg/day	1 (4.2)	5 (6.6)	0.66
2.5–3 mg/kg/day	23 (95.8)	71 (93.4)	0.66
Load of 2–2.5 mg/kg as first dose	8 (33.3)	19 (25.0)	0.42
Polymyxin B monotherapy	1 (4.2)	1 (1.3)	0.38
Concomitant with *β*-lactam	8 (33.3)	31 (40.8)	0.67
Concomitant with carbapenem	6 (25.0)	20 (26.3)	0.90
Concomitant with tigecycline	15 (62.5)	22 (29.0)	0.00
Concomitant with nephrotoxic drugs^a^	4 (16.7)	6 (7.9)	0.21
**Baseline condition (before polymyxin B treatment)**			
WBC (×10^9^/L)	12.2 ± 6.6	10.6 ± 6.7	0.30
Hemoglobin (g/L)	83.4 ± 18.1	89.2 ± 23.7	0.28
Albumin (g/L)	30.5 ± 5.4	33.9 ± 5.5	0.01
Cr (µmol/L)	162.1 ± 119.5	113.2 ± 129.7	0.10
Kidney injury	12 (45.8)	16 (21.1)	0.02
**Outcome**			
Mortality	16 (66.7)	27 (35.5)	0.01
Late nephrotoxicity (on the 7th day of polymyxin B treatment)	16/19 (84.2)	9/59 (15.3)	0.00

^a^ Includes amikacin, sulfamethoxazole and ciprofloxacin in this study. APACHE; acute physiology and chronic health evaluation), CPR; cardio-pulmonary resuscitation, ICU; (intensive care unit, CCI; Charlson comorbidity index, WBC; white blood cell, ALT; alanine aminotransferase, AST; aspartate transaminase, TBIL; total bilirubin, Cr; creatinine.

**TABLE 3:** Univariate analysis for late nephrotoxicity.

	AKI on the 7th day of treatment	Non-AKI on the 7th day of treatment	*p-*value
	N = 25 (mean ± SD, or n (%))	N = 54 (mean ± SD, or n (%))	
**Demographics**			
Age (years)	59.2 ± 17.8	58.7 ± 15.7	0.90
Gender (male)	17 (68.0)	39 (72.2)	0.70
APACHE II scores	19.0 ± 9.6	18.6 ± 8.6	0.82
CPR history	1 (4.0)	10 (18.5)	0.08
ICU Admission	22 (88.0)	47 (87.0)	0.91
Hospitalization days	31.6 ± 19.4	46.5 ± 30.1	0.03
Favorable clinical response	15 (60.0)	42 (77.8)	0.10
**Comorbidities**			
CCI	4.6 ± 3.2	4.4 ± 2.9	0.81
Malignant disease	3 (12.0)	8 (14.8)	0.74
Pulmonary diseases	4 (16.0)	11 (20.4)	0.65
Heart Diseases	12 (48.0)	20 (37.0)	0.36
Diabetes	6 (24.0)	10 (18.5)	0.57
Kidney disease	10 (40.0)	18 (33.3)	0.57
Transplantation history	5 (20.0)	11 (20.4)	0.97
Kidney transplantation	3 (12.0)	6 (11.1)	0.91
Surgery	8 (32.0)	23 (42.6)	0.37
**Polymyxin B treatment**			
Total dosage (mg)	1,530.0 ± 736.9	1,584.8 ± 1,026.8	0.00
2 mg/kg/day	11.2 ± 3.6	12.1 ± 5.4	0.43
2.5–3 mg/kg/day	2 (8.0)	4 (7.4)	0.93
Load of 2–2.5 mg/kg as first dose	23 (92.0)	50 (92.6)	0.93
Polymyxin B monotherapy	1 (4.0)	1 (1.9)	0.57
Concomitant with *β*-lactam	10 (40.0)	20 (37.0)	0.80
Concomitant with carbapenem	5 (20.0)	18 (33.3)	0.23
Concomitant with tigecycline	14 (56.0)	15 (27.8)	0.02
Concomitant with nephrotoxic drugs^a^	5 (20.0)	5 (9.3)	0.18
**Baseline condition (before polymyxin B treatment)**			
WBC (×10^9^/L)	14.2 ± 9.0	10.4 ± 5.5	0.02
Hemoglobin (g/L)	86.6 ± 19.1	88.8 ± 23.9	0.69
Albumin (g/L)	30.7 ± 5.7	34.1 ± 5.1	0.01
Cr (µmol/L)	135.7 ± 96.8	127.6 ± 139.6	0.79
Kidney injury	9 (36.0)	14 (25.9)	0.36
Early nephrotoxicity (on the 3rd day of polymyxin B treatment)	16/25 (64.0)	3/53 (5.7)	0.00
**Outcome**			
Mortality	16 (64.0)	13 (24.1)	0.00

^a^ Includes amikacin, sulfamethoxazole and ciprofloxacin in this study. APACHE; acute physiology and chronic health evaluation, CPR; cardio-pulmonary resuscitation, ICU; intensive care unit, CCI; Charlson comorbidity index, WBC; white blood cell, ALT; alanine aminotransferase, AST; aspartate transaminase, TBIL; total bilirubin, Cr; creatinine.

### Analysis of Clinical Responses

We compared the distribution of various characteristics including demographics, comorbidities, underlying conditions, concomitant antibiotic therapy, pathogens, and adverse events, between groups with clinically favorable and unfavorable outcomes (Table 1). Univariate analysis revealed a favorable clinical outcome in female patients, patients where the polymyxin B minimum inhibitory concentration (MIC) of the infecting organism was ≤0.5 mg/L, patients with favorable microbiologic response or patients with reduced APACHE II scores. Conversely, patients with an unfavorable clinical outcome tended to have hematological disease or cardio-pulmonary resuscitation (CPR) histories or be infected with a greater number of pathogens (bacteria and fungi). Overall, patients with a favorable clinical response had reduced all-cause mortality without a significant difference in nephrotoxicity.

Backward logistic regression analysis showed that the following parameters were independently associated with favorable clinical outcomes: APACHE II scores (odds ratio (OR) = 0.89, 95% confidence interval (CI) 0.82–0.97; *p* = 0.01), CPR history (OR = 0.09, 95% CI 0.01–0.80, *p* = 0.03), numbers of pathogens per patient (OR = 0.05, 95% CI 0.01–0.25, *p* = 0.00), and a favorable microbiological response (OR = 23.44, 95% CI 5.26–104.41; *p* = 0.00) (Table 4).

**TABLE 4:** Multivariate analysis of a favorable clinical outcome.

Variable	Or (95%CI)	*p*-value
APACHE II scores	0.89 (0.82–0.97)	0.01
CPR history	0.09 (0.01–0.80)	0.03
Numbers of pathogens per patient	0.05 (0.01–0.25)	0.00
Favorable microbiological response	23.44 (5.26–104.41)	0.00
Platelets before polymyxin B treatment (×10^9^/L)	1.01 (1.00–1.02)	0.00
TBIL before polymyxin B treatment (µmol/L)	1.02 (1.00–1.03)	0.01

APACHE; acute physiology and chronic health evaluation, CPR; cardio-pulmonary resuscitation, TBIL; total bilirubin, Cr; creatinine.

### Nephrotoxicity Analysis

#### Early Nephrotoxicity Analysis

The characteristics of patients with AKI (n = 24) and non-AKI (n = 76) following 3 days of polymyxin B treatment were analyzed (Table 2). Those with early nephrotoxicity on the third day tended to receive a higher total dose of polymyxin B and concomitant tigecycline treatment, and had reduced baseline albumin levels (30.5 ± 5.4 g/L vs. 33.9 ± 5.5 g/L, *p* = 0.01) and pre-existing kidney injury. Outcomes analysis showed that patients with early nephrotoxicity had increased all-cause mortality and an increased prevalence of late nephrotoxicity. Backward logistic regression showed that higher baseline albumin (OR = 0.84, 95% CI 0.74–0.94, *p* = 0.00) and concomitant treatment with tigecycline (100–200 mg/day; OR = 6.75, 95% CI 2.05–22.23, *p* = 0.00) were independently associated with early nephrotoxicity (Table 5).

**TABLE 5:** Multivariate analysis of early nephrotoxicity.

Variable	Or (95%CI)	*p*-value
Total dose of polymyxin B	1.00 (1.00–1.00)	0.06
Concomitant with tigecycline	6.75 (2.05–22.23)	0.00
Albumin before polymyxin B treatment (g/L)	0.84 (0.74–0.94)	0.00
Kidney injury before polymyxin B treatment (µmol/L)	2.76 (0.92–8.29)	0.07

#### Late Nephrotoxicity Analysis

We also examined the characteristics of patients with AKI (n = 25) and non-AKI (n = 54) following 7 days of polymyxin B treatment (Table 3). Late nephrotoxicity occurred more frequently in patients who had been hospitalized for longer, who received a higher total polymyxin B dose or concomitant tigecycline treatment, had higher baseline WBC counts or reduced baseline albumin levels, were infected with *Escherichia coli* or who had experienced nephrotoxicity following 3 days of polymyxin B therapy (i.e. early nephrotoxicity). Interestingly, there were no significant differences in the baseline kidney injury characteristics between patients with and without late nephrotoxicity. Similar to those with early nephrotoxicity, patients with late nephrotoxicity had a higher all-cause mortality than those who did not develop nephrotoxicity. Furthermore, backward logistic regression showed that nephrotoxicity on day three of polymyxin B treatment (OR = 39.43, 95% CI 7.64–203.62, *p* = 0.00) was independently associated with late nephrotoxicity (Table 6).

**TABLE 6:** Multivariate analysis of late nephrotoxicity.

Variable	Or (95%CI)	*p-*value
Hospitalization days	0.97 (0.94–1.00)	0.07
Nephrotoxicity on the 3rd day of polymyxin B treatment	39.43 (7.64–203.62)	0.00
WBC before polymyxin B treatment (×10^9^/L)	1.08 (0.99–1.19)	0.08

WBC; white blood cell.

## Discussion

Over the last decade, polymyxins have become a last-line treatment for nosocomial pneumonia caused by MDR, XDR or PDR bacteria. A limited number of studies have discussed the effectiveness and safety of polymyxin B in European and American patients (Furtado et al., 2007; Nelson et al., 2015; John et al., 2018). To optimize the clinical use of polymyxin B in Chinese patients, we conducted this retrospective study to identify risk factors for favorable outcomes and predictive factors for nephrotoxicity. As many patients had severe comorbidities, we categorized patients based on clinical response rather than all-cause mortality. Neurotoxicity was not discussed in this work because the majority of patients underwent mechanical ventilation with sedation which made assessing neurotoxicity difficult.

Most (94.4%) patients received a dose of polymyxin B of between 2.5 and 3 mg/kg/day, based on the results from a number of previous studies (Nelson et al., 2015; Rigatto et al., 2015) and international consensus guidelines (Tsuji et al., 2019) that have used or recommend a dose of polymyxin B in the range of 2.5–3 mg/kg/day. The relatively narrow range of polymyxin B daily dose reduced sample differences and increased the reliability of our results. Current animal PK/pharmacodynamics (PD) studies (Cheah et al., 2015; Landersdorfer et al., 2017) indicate the currently recommended polymyxin B dosage regimens do not achieve bacterial stasis in lung infections caused by Gram-negative bacteria, suggesting polymyxins might not be ideal for the treatment of pneumonia. However, our study and a Brazilian study (Furtado et al., 2007) both show that intravenous polymyxin B can lead to favorable clinical and microbiological outcomes in pneumonia patients. The reason for the difference between patients and animal models might be different infection pathologies. Unlike pneumonia patients who were infected directly in the lung tissue, mouse models involve spraying bacterial suspension into the trachea (Cheah et al., 2015; Landersdorfer et al., 2017). Further clinical studies on polymyxin B PK/PD in pneumonia patients are urgently needed.

Consistent with previous studies (Furtado et al., 2007; Falagas et al., 2010), a high APACHE II score and CPR history were predictive of an unfavorable clinical response to polymyxin B therapy. As the clearance and plasma concentration of polymyxin B are not affected by APACHE II scores (Sandri et al., 2013), our findings reveal that the severity of disease is directly related to clinical outcomes. The regression model also showed that pneumonia was more frequently cured in patients with reduced numbers of causative pathogens. Our results confirm that polymicrobial infections might lead to polymyxin B treatment failure in some patients. Although the duration of polymyxin B treatment was removed in the regression models, univariate analysis demonstrated that patients with favorable outcomes had increased polymyxin B treatment durations (11.3 ± 5.6 days). This result is at odds with guidelines for HAP/VAP (Kalil et al., 2016) that recommended a 7-days course of antimicrobial therapy for HAP (non-VAP). The reason for this difference might be that patients in our study included both HAP and VAP patients with severe comorbidities and causative pathogens that were drug-resistant. On the other hand, the shorter duration of polymyxin B treatment in unfavorable group could not exclude the influence of early mortality of severe cases.

Furthermore, we observed that a favorable microbiological response was most directly and independently associated with a favorable clinical outcome. In addition, the proportion of patients with a MIC < 0.5 mg/L was significantly greater in the group that had a favorable clinical response than in the group with unfavorable clinical response (59.7% vs. 37.5%). In addition, we observed no significant difference in the age of patients exhibiting different clinical outcomes, indicating that the effectiveness of polymyxin B might not be problematic in elderly patients without severe underlying conditions.

Notably, our study contributes to the understanding of polymyxin B-induced nephrotoxicity by assessing the condition at different time points, specifically, following three and 7 days of polymyxin B treatment. Although the clearance of polymyxin B does not depend on glomerular filtration, in critically ill patients 90–95% of filtered polymyxin B is reabsorbed by tubular cells (Sandri et al., 2013; Zavascki et al., 2017). Mohammad et al. detected (Azad et al., 2015) polymyxin concentrations in human kidney tubular cells ∼4,760-fold higher than extracellular concentrations, indicating an extraordinary intracellular accumulation of polymyxin within these cells. Because of the reabsorption process and intracellular accumulation of polymyxins, tubular cells are exposed to high concentrations of polymyxins which can directly cause damage (Abdelraouf et al., 2012; Sandri et al., 2013). This process might explain how polymyxin B causes damage to the kidney despite its non-renal drug clearance. Previous studies have reported that the prevalence of nephrotoxicity due to polymyxin B ranged from 10.0 to 23.1% (Ouderkirk et al., 2003; Sobieszczyk et al., 2004; Phe et al., 2014). However, different definitions of nephrotoxicity were applied and a wide range of polymyxin B doses administered (12–225 mg/day), which may explain the different results in previous studies. Despite using the same RIFLE criteria (Kellum et al., 2008) to define nephrotoxicity as used in the present study, Kady et al. (Phe et al., 2014) reported a lower incidence of polymyxin B-associated nephrotoxicity than we observed (23.1% vs. 31.6%). The higher doses of polymyxin B administered in our study than in Kady’s (2.63 ± 0.5 vs. 1.5 ± 0.5 mg/kg/day) may explain the higher prevalence of nephrotoxicity.

Several studies suggested that preexisting renal dysfunction prior to polymyxin B treatment may be a risk factor for nephrotoxicity (Crass et al., 2017; John et al., 2018), although it is unclear whether this renal dysfunction is a baseline condition or is caused by a severe infection. Indeed, infection-induced renal dysfunction might be temporary and reversed by adequate antibiotic treatment, yet physicians may be hesitant to use salvageable polymyxin B under such conditions. Our results indicate that the presence of renal dysfunction prior to the commencement of polymyxin B therapy was not associated with nephrotoxicity on the seventh day of polymyxin B administration. This finding may provide physicians with confidence to use intravenous polymyxin B in a timely manner irrespective of the initial renal function. Moreover, decreasing the dose of polymyxin B in patients experiencing renal injury may lead to suboptimal plasma exposure with potentially adverse consequences on clinical and microbiological outcomes, as well as on the development of resistance (Elias et al., 2010; Abdelraouf et al., 2012; Sandri et al., 2013).

To achieve optimal clinical outcome, guidelines for HAP and VAP (Kalil et al., 2016) strongly recommend a seven-day course of antimicrobial therapy. However, 48 h of antibiotic treatment may be inadequate to bring an infection under control by the third day. Given the difficulties in differentiating between drug-associated AKI and infection-induced AKI, it is possible nephrotoxicity observed following 3 days of polymyxin B treatment is incorrectly attributed to the polymyxin rather than the infecting organism. Conversely, intracellular accumulation of and exposure to polymyxin B increases with increasing duration of therapy. The above reasons could explain the different incidences of nephrotoxicity at different time points. Additionally, early nephrotoxicity on the third day of polymyxin B treatment was found to be a promising and reliable predictive factor for later nephrotoxicity, most likely due to potential accumulation of polymyxin B in tubular cells. When nephrotoxicity is experienced after 3 days of therapy, caution is required if polymyxin B treatment is to be continued and close monitoring of kidney function is recommended.

Finally, our study revealed some other interesting findings about nephrotoxicity in Chinese patients. In patients with both early and late nephrotoxicity, albumin levels (30.5 ± 5.4 and 30.7 ± 5.7 g/L, respectively) were reduced, and the prevalence of concomitant treatment with tigecycline was increased. These results suggest that reduced albumin levels and concomitant tigecycline treatment may increase polymyxin B-associated nephrotoxicity. Given that polymyxin B binds to plasma proteins with a percentage range from 82.3 ± 4.30% to 91.4 ± 1.65% (Landersdorfer et al., 2017; Sivanesan et al., 2017), a reduction in the concentration of serum albumin might lead to an increased concentration of unbound drug and greater tubular reabsorption. Further studies are required to assess the PK/PD/toxicodynamics of polymyxin B in different types of patients, including those with hypoproteinemia and tigecycline treatment.

## Conclusion

Collectively, our findings support the use of polymyxin B to treat pneumonia caused by MDR and XDR Gram-negative pathogens. The severity of disease and polymicrobial infections were risk factors for a poor clinical outcome. Nephrotoxicity that occurred following 3 days of polymyxin B treatment (early nephrotoxicity) was a reliable risk factor for late nephrotoxicity. Future well-designed, controlled trials are warranted to investigate the effectiveness and safety of polymyxin B in patients.

## Data Availability Statement

Data are available related to this study can be accessible from chenyan99727@csu.edu.cn with reasonable requests.

## Ethics Statement

This study was approved and supervised by the Medical Research Ethics Committee of the Second Xiangya Hospital, Central South University (LYF2020059). Written informed consent was waived.

## Author Contributions

All authors listed have made a substantial, direct, and intellectual contribution to the work and approved it for publication.

## Funding

This study was supported by the National Key Clinical Specialist Construction Programs of China ((2012) No. 650); the National Natural Science Foundation of China (81,400,032); the Natural Science Foundation of Hunan Province (2019JJ50877); and the Fundamental Research Funds for the Central universities of Central South university (2020zzts879).

## Conflict of Interest

The authors declare that the research was conducted in the absence of any commercial or financial relationships that could be construed as a potential conflict of interest.

## References

[B1] Abdelraouf K. Braggs K. H. Yin T. Truong L. D. Hu M. Tam V. H. (2012). Characterization of polymyxin B-induced nephrotoxicity: implications for dosing regimen design. Antimicrob. Agents Chemother. 56, 4625–4629. 10.1128/AAC.00280-12 22687519 PMC3421883

[B2] Azad M. A. K. Roberts K. D. Yu H. H. Liu B. Schofield A. V. James S. A. (2015). Significant accumulation of polymyxin in single renal tubular cells: a medicinal chemistry and triple correlative microscopy approach. Anal. Chem. 87, 1590–1595. 10.1021/ac504516k 25553489 PMC4318625

[B3] Charlson M. Szatrowski T. P. Peterson J. Gold J. (1994). Validation of a combined comorbidity index. J. Clin. Epidemiol. 47, 1245–1251. 10.1016/0895-4356(94)90129-5 7722560

[B4] Cheah S.-E. Wang J. Nguyen V. T. Turnidge J. D. Li J. Nation R. L. (2015). New pharmacokinetic/pharmacodynamic studies of systemically administered colistin against *Pseudomonas aeruginosa* and Acinetobacter baumannii in mouse thigh and lung infection models: smaller response in lung infection. J. Antimicrob. Chemother. 70, 3291–3297. 10.1093/jac/dkv267 26318190

[B5] Crass R. L. Rutter W. C. Burgess D. R. Martin C. A. Burgess D. S. (2017). Nephrotoxicity in patients with or without cystic fibrosis treated with polymyxin B compared to colistin. Antimicrob. Agents Chemother. 61, e02329–16 10.1128/AAC.02329-16 28167560 PMC5365730

[B6] Elias L. S. Konzen D. Krebs J. M. Zavascki A. P. (2010). The impact of polymyxin B dosage on in-hospital mortality of patients treated with this antibiotic. J. Antimicrob. Chemother. 65, 2231–2237. 10.1093/jac/dkq285 20685752

[B7] European Committee on Antimicrobial Susceptibility Testing (2016). “Recommendations for MIC determination of colistin (polymyxin E) as recommended by the joint CLSI-EUCAST Polymyxin Breakpoints Working Group,” in European committee on antimicrobial susceptibility testing (Växjö, Sweden: European Committee on Antimicrobial Susceptibility Testing). http://www.eucast.org/fileadmin/src/media/PDFs/EUCAST_files/General_documents/Recommendations_for_MIC_determination_of_colistin_March_2016. pdf.

[B8] Falagas M. E. Kasiakou S. K. Saravolatz L. D. (2005). Colistin: the revival of polymyxins for the management of multidrug-resistant gram-negative bacterial infections. Clin. Infect. Dis. 40, 1333–1341. 10.1086/429323 15825037

[B9] Falagas M. E. Rafailidis P. I. Ioannidou E. Alexiou V. G. Matthaiou D. K. Karageorgopoulos D. E. (2010). Colistin therapy for microbiologically documented multidrug-resistant Gram-negative bacterial infections: a retrospective cohort study of 258 patients. Int. J. Antimicrob. Agents 35, 194–199. 10.1016/j.ijantimicag.2009.10.005 20006471

[B10] Furtado G. H. C. d’Azevedo P. A. Santos A. F. Gales A. C. Pignatari A. C. Medeiros E. A. (2007). Intravenous polymyxin B for the treatment of nosocomial pneumonia caused by multidrug-resistant *Pseudomonas aeruginosa* . Int. J. Antimicrob. Agents 30, 315–319. 10.1016/j.ijantimicag.2007.05.017 17631984

[B11] John J. F. Falci D. R. Rigatto M. H. Oliveira R. D. Kremer T. G. Zavascki A. P. (2018). Severe infusion-related adverse events and renal failure in patients receiving high-dose intravenous polymyxin B. Antimicrob. Agents Chemother. 62, e01617-17 10.1128/AAC.01617-17 29038262 PMC5740322

[B12] Kalil A. C. Metersky M. L. Klompas M. Muscedere J. Sweeney D. A. Palmer L. B. (2016). Management of adults with hospital-acquired and ventilator-associated pneumonia: 2016 clinical practice guidelines by the infectious diseases society of America and the American thoracic society. Clin. Infect. Dis. 63, e61–e111. 10.1093/cid/ciw353 27418577 PMC4981759

[B13] Kelesidis T. Falagas M. E. (2015). The safety of polymyxin antibiotics. Expet. Opin. Drug Saf. 14, 1687–1701. 10.1517/14740338.2015.1088520 PMC8082492 26365594

[B14] Kellum J. A. Bellomo R. Ronco C. (2008). Definition and classification of acute kidney injury. Nephron Clin. Pract. 109, c182–c187. 10.1159/000142926 18802365

[B15] Landersdorfer C. B. Wang J. Wirth V. Chen K. Kaye K. S. Tsuji B. T. (2017). Pharmacokinetics/pharmacodynamics of systemically administered polymyxin B against *Klebsiella pneumoniae* in mouse thigh and lung infection models. J. Antimicrob. Chemother. 73, 462–468. 10.1093/jac/dkx409 PMC5890666 29149294

[B16] Levin A. Stevens P. E. Bilous R. W. Coresh J. De Francisco A. L. M. De Jong P. E. (2013). ‘Kidney Disease: improving Global Outcomes (KDIGO) CKD Work Group. KDIGO 2012 clinical practice guideline for the evaluation and management of chronic kidney disease. Kidney Int. Suppl. 3, 1–150. 10.1038/kisup.2012.73 23732715

[B17] Li J. Nation R. L. Turnidge J. D. Milne R. W. Coulthard K. Rayner C. R. (2006). Colistin: the re-emerging antibiotic for multidrug-resistant Gram-negative bacterial infections. Lancet Infect. Dis. 6, 589–601. 10.1016/S1473-3099(06)70580-1 16931410

[B18] Nelson B. C. Eiras D. P. Gomez-Simmonds A. Loo A. S. Satlin M. J. Jenkins S. G. (2015). Clinical outcomes associated with polymyxin B dose in patients with bloodstream infections due to carbapenem-resistant Gram-negative rods. Antimicrob. Agents Chemother. 59, 7000–7006. 10.1128/AAC.00844-15 26324272 PMC4604419

[B19] Ouderkirk J. P. Nord J. A. Turett G. S. Kislak J. W. (2003). Polymyxin B nephrotoxicity and efficacy against nosocomial infections caused by multiresistant gram-negative bacteria. Antimicrob. Agents Chemother. 47, 2659–2662. 10.1128/aac.47.8.2659-2662.2003 12878536 PMC166058

[B20] Phe K. Lee Y. McDaneld P. M. Prasad N. Yin T. Figueroa D. A. (2014). *In vitro* assessment and multicenter cohort study of comparative nephrotoxicity rates associated with colistimethate versus polymyxin B therapy. Antimicrob. Agents Chemother. 58, 2740–2746. 10.1128/AAC.02476-13 24566187 PMC3993221

[B21] Quintanilha J. C. F. Duarte N. d. C. Lloret G. R. Visacri M. B. Mattos K. P. H. Dragosavac D. (2019). Colistin and polymyxin B for treatment of nosocomial infections in intensive care unit patients: pharmacoeconomic analysis. Int. J. Clin. Pharm. 41, 74–80. 10.1007/s11096-018-0766-x 30552622

[B22] Rello J. Van Engelen T. S. R. Alp E. Calandra T. Cattoir V. Kern W. V. (2018). Towards precision medicine in sepsis: a position paper from the European Society of Clinical Microbiology and Infectious Diseases. Clin. Microbiol. Infect. 24, 1264–1272. 10.1016/j.cmi.2018.03.011 29581049

[B23] Rigatto M. H. Behle T. F. Falci D. R. Freitas T. Lopes N. T. Nunes M. (2015). Risk factors for acute kidney injury (AKI) in patients treated with polymyxin B and influence of AKI on mortality: a multicentre prospective cohort study. J. Antimicrob. Chemother. 70, 1552–1557. 10.1093/jac/dku561 25604744

[B24] Sandri A. M. Landersdorfer C. B. Jacob J. Boniatti M. M. Dalarosa M. G. Falci D. R. (2013). Population pharmacokinetics of intravenous polymyxin B in critically ill patients: implications for selection of dosage regimens. Clin. Infect. Dis. 57, 524–531. 10.1093/cid/cit334 23697744

[B25] Sivanesan S. Roberts K. Wang J. Chea S. E. Thompson P. E. Li J. (2017). Pharmacokinetics of the individual major components of polymyxin B and colistin in rats. J. Nat. Prod. 80, 225–229. 10.1021/acs.jnatprod.6b01176 28080060

[B26] Sobieszczyk M. E. Furuya E. Y. Hay C. M. Pancholi P. Della-Latta P. Hammer S. M. (2004). Combination therapy with polymyxin B for the treatment of multidrug-resistant Gram-negative respiratory tract infections. J. Antimicrob. Chemother. 54, 566–569. 10.1093/jac/dkh369 15269195

[B27] Tacconelli E. Carrara E. Savoldi A. Harbarth S. Mendelson M. Monnet D. L. (2018). Discovery, research, and development of new antibiotics: the WHO priority list of antibiotic-resistant bacteria and tuberculosis. Lancet Infect. Dis. 18, 318–327. 10.1016/S1473-3099(17)30753-3 29276051

[B28] Thamlikitkul V. Dubrovskaya Y. Manchandani P. Ngamprasertchai T. Boonyasiri A. Babic J. T. (2017). Dosing and pharmacokinetics of polymyxin B in patients with renal insufficiency. Antimicrob. Agents Chemother. 61, e01337–16. 10.1128/AAC.01337-16 PMC5192162 27799209

[B29] Thomas V. M. Brown R. M. Ashcraft D. S. Pankey G. A. (2019). Synergistic effect between nisi and polymyxin B against prodrug-resistant and extensively drug-resistant Acinetobacter baumannii. Int. J. Antimicrob. Agents 53, 663–668. 10.1016/j.ijantimicag.2019.03.009 30880230

[B30] Tsuji B. T. Pogue J. M. Zavascki A. P. Paul M. Daikos G. L. Forrest A. (2019). International consensus guidelines for the optimal use of the polymyxins: endorsed by the American college of clinical pharmacy (ACCP), European society of clinical microbiology and infectious diseases (ESCMID), infectious diseases society of America (IDSA), international society for anti‐infective Pharmacology (ISAP), society of critical care medicine (SCCM), and society of infectious diseases pharmacists (SIDP). Pharmacotherapy 39, 10–39. 10.1002/phar.2209 30710469 PMC7437259

[B31] Zavascki A. P. Goldani L. Z. Li J. Nation R. L. (2007). Polymyxin B for the treatment of multidrug-resistant pathogens: a critical review. J. Antimicrob. Chemother. 60, 1206–1215. 10.1093/jac/dkm357 17878146

[B32] Zavascki A. P. Nation R. L. (2017). Nephrotoxicity of polymyxins: is there any difference between colistimethate and polymyxin B? Antimicrob. Agents Chemother. 61, e02319–16. 10.1128/AAC.02319-16 27993859 PMC5328560

